# Raman Enhancement and Photo-Bleaching of Organic Dyes in the Presence of Chemical Vapor Deposition-Grown Graphene

**DOI:** 10.3390/nano7100337

**Published:** 2017-10-19

**Authors:** Jiaxin Weng, Shichao Zhao, Zhiting Li, Karen B. Ricardo, Feng Zhou, Hyojeong Kim, Haitao Liu

**Affiliations:** 1 College of Materials & Environmental Engineering, Hangzhou Dianzi University, Hangzhou 310018, China; weng_jiaxin@126.com; 2 Department of Chemistry, University of Pittsburgh, Pittsburgh, Pennsylvania, PA 15260, USA; zhiting46@gmail.com (Z.L.); kbr6@pitt.edu (K.B.R.); zfedward1988@gmail.com (F.Z.); hyk31@pitt.edu (H.K.)

**Keywords:** graphene, h-BN, dye, photo-bleaching, photo-stability, kinetics

## Abstract

Fluorescent organic dyes photobleach under intense light. Graphene has been shown to improve the photo-stability of organic dyes. In this paper, we investigated the Raman spectroscopy and photo-bleaching kinetics of dyes in the absence/presence of chemical vapor deposition (CVD)-grown graphene. We show that graphene enhances the Raman signal of a wide range of dyes. The photo-bleaching of the dyes was reduced when the dyes were in contact with graphene. In contrast, monolayer hexagonal boron nitride (h-BN) was much less effective in reducing the photo-bleaching rate of the dyes. We attribute the suppression of photo-bleaching to the energy or electron transfer from dye to graphene. The results highlight the potential of CVD graphene as a substrate for protecting and enhancing Raman response of organic dyes.

## 1. Introduction

It is generally known that organic dyes degrade upon intense light exposure. During trace analysis by Raman spectroscopy, dyes need to be exposed to an intense laser for several to several tens of minutes. Improving the photo-stability of dye will significantly improve the sensitivity of Raman-based sensing methods. Graphene, with a zero energy gap, can participate in both energy and electron transfer with organic dyes. Many studies reported the interaction between graphene and organic dyes, focusing on the impact of graphene on the photophysics of the dye. 

A number of studies have investigated the fluorescence quenching of organic dyes in the presence of exfoliated graphene and suggested that the quenching mechanism was energy or electron transfer between the dye and graphene [1]. Swathi et al. studied the energy transfer from dye to graphene. They performed calculations using both Dirac cone approximation and the tight-binding model for graphene and obtained the exponential distance dependence of the rate of nonradioactive energy transfer from an excited dye molecule to graphene [2]. Xie et al. reported the interaction between graphene and fluorescent dyes rhodamine 6G and protoporphyrin IX. They observed that the fluorescence intensity was suppressed and the Raman/fluorescence intensity ratio was increased when the dye was in contact with graphene. They proposed that the observations were mainly due to the graphene-induced fluorescence quenching [3]. Dantham et al. investigated the fluorescence quenching of fluorescein 27 dye in the presence of graphene. They proposed that a complex is formed via π-π stacking between the dye molecule and graphene, through which the charge transfer occurred [4]. Similar research was conducted by Pang et al., in which they observed a dynamic quenching of cationic dye and concluded that the electron transfer is responsible for the quenching [5]. Xiong et al. investigated the photocatalytic degradation of rhodamine B (RhB) dye over graphene-gold nanocomposites. They suggested that the electron transfer from excited dye to graphene reduces the degradation of dye [6]. The same electron transfer pathway was mentioned by Zhuang et al. [7].

The fluorescence quenching increases the ratio of Raman intensity to that of fluorescence. Thus graphene can be used as a substrate for Raman Spectroscopy. Early studies showed that exfoliated graphene enhances the Raman intensity of some fluorescence dyes (e.g., rhodamine (R6G) and protoporphyrin (PPP)) [3]. The degree of Raman enhancement depends on both the electronic energy level alignment between the dye and graphene and the symmetry of the dye molecule, indicating that charge transfer is responsible for the enhancement [8]. These early studies used high quality, almost defect-free exfoliated graphene. Later, chemical vapor deposition (CVD)-grown graphene was also used and similar results were reported. CVD-grown graphene has a much higher density of defects and more corrugations than exfoliated graphene [9]. These differences in the structure may also result in different behaviors in Raman enhancement. For example, Xu et al. prepared copper nanoparticles encapsulated with CVD-grown graphene and demonstrated Raman-based detection of adenosine in serum at 5 nM concentration [10]. Recently, Feng et al. prepared nitrogen-doped graphene by CVD and showed that the presence of nitrogen can further enhance the Raman response of adsorbed dye [11].

A much less appreciated benefit of using graphene substrate is its enhancement of the photochemical stability of organic dyes. The mechanism of such an effect was generally attributed to the barrier property of graphene. Zhao et al. studied the surface-enhanced Raman spectroscopy of R6G dyes sandwiched between CVD-grown graphene and the magnetron sputtered Ag film. They observed that the photo-stability of the dye was increased in the presence of graphene. They suggest that the graphene acts as a barrier film preventing the reaction between the oxygen in the air and the dye underneath graphene [12]. Xu et al. studied the Raman spectra of Copper(II) phthalocyanine (CuPc) absorbed on the surface of graphene/metal nanoparticles. They found the photo-stability of CuPc increased and suggested that the increase of the photo-stability may be caused by the π-π interaction between CuPc and graphene [13].

Here we showed that CVD-grown graphene enhances the Raman signals of a wide range of dyes including fluorescein isothiocyanate (FITC), pyrromethene, coumarin 540A, fluorescein 548, disodium fluorescein, and kiton red 620, suggesting a broad application of CVD-grown graphene in Raman enhancement. We also show that the fluorescence was quenched and the photo-bleaching rate was significantly slowed down by 46.3% in the presence of graphene. 

## 2. Experimental

### 2.1. Chemicals

Silicon wafer with 300 nm of oxide layer was purchased from Universitywafer (Boston, MA, USA), Pyrromethene, coumarin 540A, fluorescein 548, disodium fluorescein, and kiton red 620 were purchased from Exciton (West Chester, OH, USA). FITC and (3-Aminopropyl)triethoxysilane (APTES) were purchased from Sigma-Aldrich (St. Louis, MO, USA). The silicon substrate was cleaned with hot piranha solution (7:3 concentrated H_2_SO_4_:35% H_2_O_2_). *Warning: Piranha is highly reactive. Avoid contact with large amount of organic materials. Work in a chemical hood and use full personal protective equipment.*

### 2.2. Preparation of CVD-Grown Single Layer Graphene

The single layer graphene used in this study was prepared by a copper-catalyzed chemical vapor deposition using methane as the carbon source [9,14,15]. Our previous work has reported a detailed characterization of the graphene sample [9]. After the synthesis, the graphene-copper substrate was coated with a thin layer of poly(methyl methacrylate) (PMMA) and the copper was etched away using a FeCl_3_ solution. The PMMA/graphene film was transferred onto a silicon wafer with or without the dye absorbed on the surface; the PMMA polymer was removed with an acetone wash to leave the graphene on the silicon wafer [16].

### 2.3. Preparation of CVD-Grown Single Layer h-BN

The single layer h-BN used in this study was prepared by copper-catalyzed CVD using Borane-ammonia complex as the h-BN source [17,18]. The Cu substrate was put in the center of tube furnace and heated to 1000 °C from room temperature in 40 min. The precursor was placed in a glass tube, which was connected to the gas line through a T-adapter at the upper stream of the Cu substrate. The precursor was heated to 100 °C from room temperature in 15 min by a water bath. The vapor of the precursor was introduced into the furnace by an Ar/H_2_ flow (Ar 4 sccm, H2 0.8 sccm). The water bath was kept at 100 °C for 15 min before the furnace was turned off and cooled from 1000 °C to room temperature under Ar/H_2_ flow. After the synthesis, the h-BN-copper substrate was coated with a thin layer of PMMA and the copper was etched away using a FeCl_3_ solution. The PMMA/h-BN film was transferred onto a silicon wafer with or without the dye absorbed on the surface; the PMMA polymer was removed with an acetone wash to leave the h-BN on the silicon wafer. 

### 2.4. Binding of APTES onto the SiO_2_ Surface

Binding of APTES was carried out by immersion of the silicon substrate into a 10 mM solution of the APTES in toluene (spectroscopic grade) [19]. After 20 min, the sample was removed from the solution and rinsed in ultrasonic baths of toluene, dichloromethane, and ethanol for 10 min, respectively.

### 2.5. Covalent Binding of FITC to an APTES-Functionalized Silicon Substrate

Covalent of FITC on the APTES-functionalized silicon substrate was carried out by immersion of the substrate in a 0.2 mM solution of FITC in ethanol for 3 h then washed in ultrasonic baths of ethanol for 10 min and acetone for 20 min–27 h. The FITC is covalently linked to the APTES through the formation of a thiourea linkage [19].

### 2.6. Absorption of Dyes to an APTES-Functionalized Silicon Substrate

Absorption of pyrromethene, coumarin 540A, fluorescein 548, disodium fluorescein, and kiton red 620 on the APTES-functionalized silicon substrate was carried out by immersion of the substrate in a 0.2 mM solution of the corresponding dye in ethanol for 3 h then washed in ultrasonic baths of ethanol for 10 min and acetone for 90 min–6 h. The washing steps are intended to remove dyes in the state of agglomerated particles or multilayer adsorption, which may not form intimate contact with the graphene and complicates the data analysis. 

### 2.7. Absorption of FITC to Silicon Substrate or CVD-Grown Graphene Substrate

Absorption of FITC on the silicon substrate with oxide layer was carried out by immersion of the substrate in a 0.2 mM solution of FITC in ethanol for 3 h then washed in ultrasonic baths of ethanol for 10 min and acetone for 20 min. For the CVD-grown graphene substrate, 0.1 mM FITC dye solution in ethanol was spin-coated on the substrate at 3250 rpm.

### 2.8. Transfer of the CVD-Grown Graphene/h-BN onto the Surface of Dye Layer

FITC, pyrromethene, coumarin 540A, fluorescein 548, disodium fluorescein, orkiton red 620 was first absorbed on the silicon substrate or PTES-functionalized silicon substratewith the method mentioned above. Then, the CVD-grown graphene or h-BN was transferred onto the substrate with the help of PMMA and water [16]. Details as below: After the synthesis, the copper-supported graphene or h-BN substrate was coated with a thin layer of PMMA and the copper was etched away using a FeCl_3_ solution. The PMMA/graphene(h-BN) film was washed with D.I. water and kept floating on the water surface with graphene side facing downward. Then, the substrate with the dye layer facing upward was dipped into the water and the PMMA/graphene(h-BN) film was picked up. After drying in air for 30 min, the PMMA polymer was removed in acetone for 20 min~12 h to leave the graphene or h-BN on the surface of the dye layer, forming a Gr(or h-BN)/dye/SiO_2_/Si structure (Gr: graphene).

### 2.9. Characterization

Fluorescence measurement: Fluorescent spectra were collected on a custom-built set-up that consisted of a 532 nm single-longitudinal-mode solid-state laser, a 532 nm clean-up filter (Semrock, Rochester, NY, USA), an inverted microscope (Nikon Eclipse Ti/U, Nikon, Minato, Tokyo, Japan), a 532 nm long-pass edge filter (Semrock), and a single-stage spectrograph (Shamrock 303, focal length: 303mm, Andor, Belfast, UK) with an attached back-illuminated charge-coupled device (CCD) camera thermoelectrically cooled to −55 °C (AndoriDus, Andor). A 40× objective (NA: 0.60) was used in all of the micro-Raman experiments. Laser power was kept below 2 mW at the sample to avoid laser-induced heating and damage to the dye and graphene. The laser power was measured by using a power meter (Fieldmaster, Coherent, Santa Clara, CA, USA).

## 3. Results and Discussion

### 3.1. Raman Enhancement by CVD Graphene

**Figure 1:**
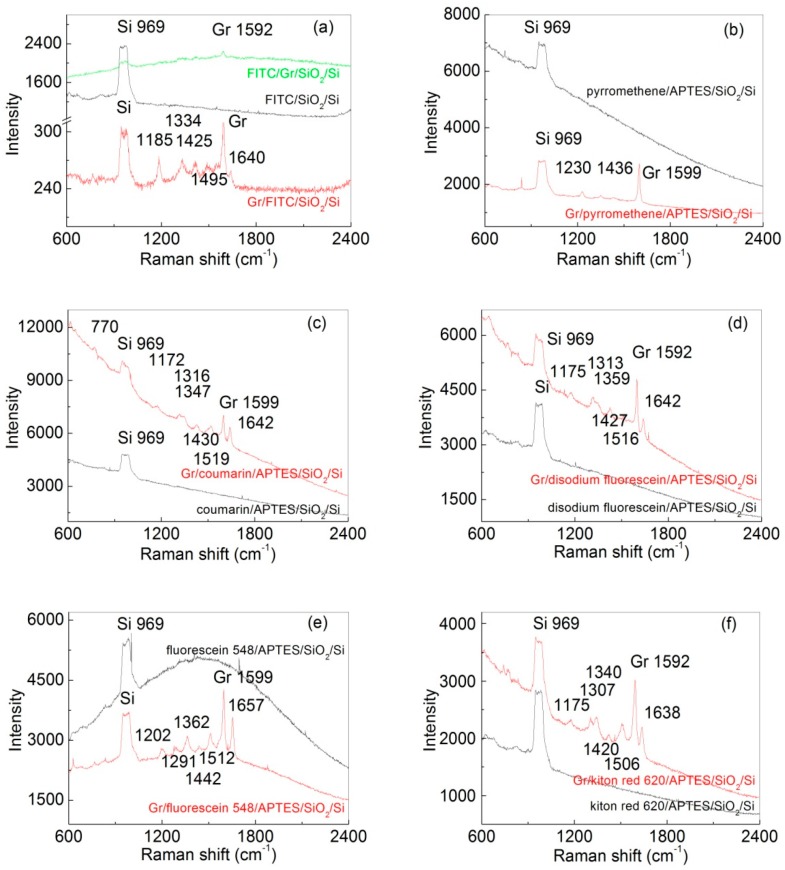
Raman-FL spectra of fluorescein isothiocyanate (FITC) (**a**), pyrromethene (**b**), coumarin (**c**), disodium fluorescein (**d**), fluorescein 548 (**e**), and kiton red 620 (**f**) covered with (red) or without graphene (black). The substrates are SiO_2_/Si in (**a**) and APTES/SiO_2_/Si in (**b**–**f**). APTES: aminopropyltriethoxysilane. For the green curve in (**a**), the SiO_2_/Si substrate was first covered with a single layer graphene forming a Gr/SiO_2_/Si structure (Gr: graphene), then the FITC dyes were adsorbed above graphene. The Raman integration time was 30 s (red and green spectra in (**a**)), 10 min (black spectrum in (**a**)), or 20 min (for (**b**–**f**)). The peaks at 969 cm^−1^ and ca. 1590–1599 cm^−1^ were from Si and graphene, respectively.

We use Raman spectroscopy to characterize the fluorescence and chemical stability of the dye. The single layer graphene sample was synthesized using a copper-catalyzed CVD method and was extensively characterized in our previous study [9]. The atomic force microscopy (AFM) image and Raman spectrum of the graphene sample can be found in Figures S1 and S2. To limit laser-induced heating and damage to the dye and graphene, the laser power was kept below 2 mW. Figure 1a shows Raman spectra of FITC in the presence and absence of CVD-grown single layer graphene. Raman peaks from both graphene (ca. 1592 cm^−1^) and FITC (1185 cm^−1^, 1334 cm^−1^, 1425 cm^−1^, 1495 cm^−1^, and 1640 cm^−1^) were clearly observed on Gr/FITC sample (Gr: graphene), while the FITC sample in the absence of graphene barely showed any peak except the Si-Si overtone (969 cm^−1^) from the Si substrate [9,20,21]. Similar results were also observed on other fluorescence dyes, such as pyrromethene, coumarin 540A, fluorescein 548, disodium fluorescein, and kiton red 620. These results clearly indicate that the Raman enhancement induced by graphene is broadly applicable for many organic dyes.In addition, we compared the Raman signal of the FITC presented above and below the graphene (Figure 1a and Figures S3) and found that Raman signal of the dye was most intensive when the dye was covered with graphene. However, the result should be interpreted with caution because the amount of dye adsorbed on the surface is likely very different in the two samples. 

Note that in Figure 1a,b,e the fluorescence intensity of dye in the absence of graphene is much higher than that in the presence of graphene; the opposite effectwas found in Figure 1c,d,f. We suspect that this inconsistent behavior is due to the different amount of dye absorbed on the substrate. Because the quenching efficiency of graphene is distance-dependent, incomplete quenching could be observed in the case of multilayer dye adsorption. It is worth noting that FITC was covalently adsorbed onto SiO_2_ via APTES, ensuring that no more than a monolayer of FITC is present. For this reason, we chose FITC in our kinetic studies of photobleaching (see below). 

### 3.2. Photo-Bleaching Kinetic of FITC Dye in the Presence of Graphene

We prepared two sets of samples with different structures: FITC/APTES/SiO_2_/Si (FITC was adsorbed on aminopropyltriethoxysilane (APTES) modified SiO_2_ surface) and Gr/FITC/APTES/SiO_2_/Si (FITC was sandwiched between graphene and the APTES modified SiO_2_/Si substrate), to compare the photochemical stability of FITC in the absence and presence of graphene. We attached FITC through APTES to the SiO_2_/Si substrate to achieve a high surface coverage of the dye through the reaction between the isothiocyanate group of the FITC and the amine group of the APTES, which forms a covalent thiourea linkage [19]. By using extensive washing, we remove the dyes that are non-covalently adsorbed to the surface. Multi-layer and aggregation of dyes could complicate our data analysis, because in such samples not all dye molecules are in intimate contact with graphene. 

**Figure 2:**
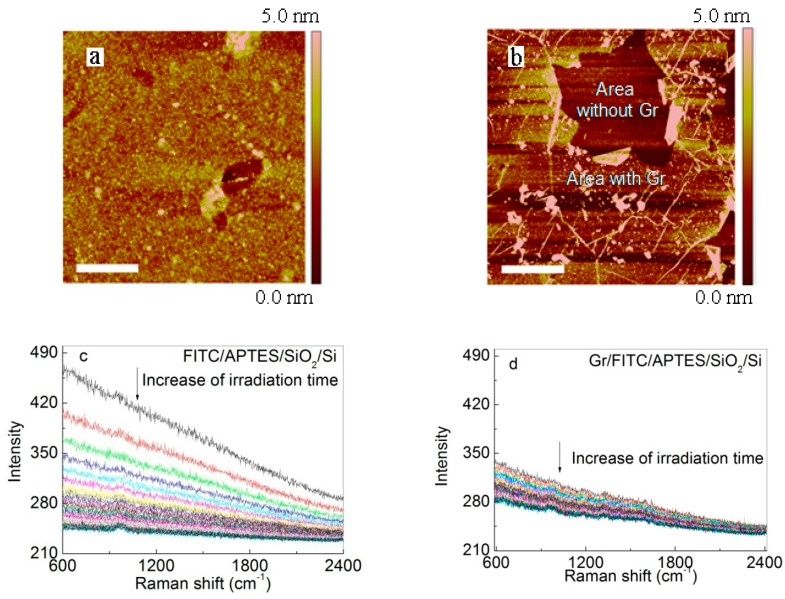
Typical (top) atomic force microscopy (AFM) images and (bottom) Raman-fluorescence spectra of FITC in the absence/presence of graphene (Gr): (**a**) FITC/APTES/SiO_2_/Si, washed in acetone for 27 h; (**b**) Gr/FITC/APTES/SiO_2_/Si, washed in acetone for 12 h. The time evolution of the fluorescence spectra (**c**,**d**) is used to quantify the photo-bleaching kinetics of the dye. The spectrum was integrated for 5 s, with continuous laser exposure and 5 s delay between spectrum acquisitions. Scale bars in the AFM images represent 500 nm in (**a**) and 1 μm in (**b**).

Figure 2 shows AFM images of these two samples. The root-mean-square (RMS) surface roughness over 2 μm × 2 μm of FITC/APTES/SiO_2_/Si was 0.60 nm. The RMS roughness of Gr/FITC/APTES/SiO_2_/Si at location free of PMMA residues (white particles) was 0.48 nm. The smooth surface in both cases indicates minimal aggregation of the dye molecule. In Figure 2b, we intentionally selected an area with a broken graphene to show the topography contrast. Note that the line features and small particles on graphene are wrinkles and poly-methylmethacrylate (PMMA) polymer residue from the graphene transfer, respectively.

**Figure 3:**
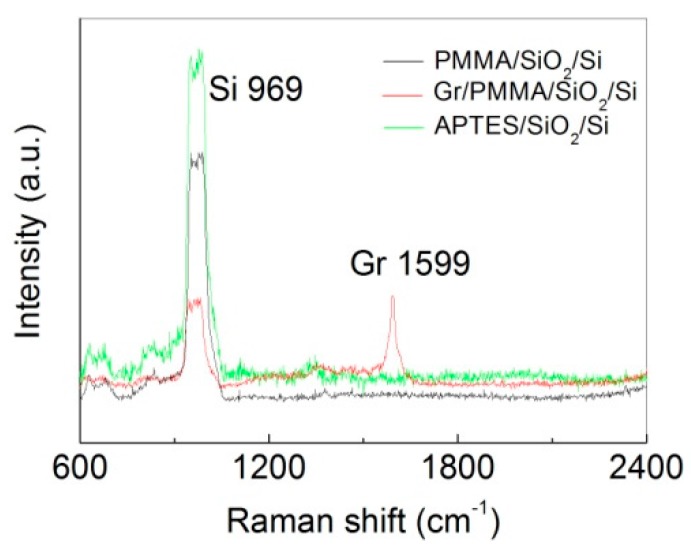
Raman spectra of poly-methylmethacrylate (PMMA) (black), graphene (red), and APTES (green), respectively. PMMA was spin-coated on SiO_2_/Si substrate (PMMA/SiO_2_/Si). Graphene (Gr) with PMMA was transferred onto the SiO_2_/Si substrate (Gr/PMMA/SiO_2_/Si). APTES was covalently attached onto the SiO_2_/Si substrate (APTES/SiO_2_/Si). The Raman integration time is ca. 2~5 min. PMMA, graphene, and APTES showed no fluorescence. Note that compared to Figure 2, the Raman peaks of Si and graphene are visible in this case due to the absence of fluorescence background and hence much reduced shot noise. The Raman peaks of PMMA and APTES were not observed due to their low Raman cross section.

Figure 2c,d displays the Raman/fluorescence spectra as a function of laser exposure time. In this experiment, we used very short integration time to improve time resolution, and hence the Raman peaks are within the noise level. For example, decent Raman spectrum can be obtained using an integration time of 10 min (Figure S4). However, with an integration time of 30 s, the individual Raman peaks were buried in the noise and can not be used to extract the photo-bleaching kinetics. To solve the problem, we note that photo-bleaching will result in the same degree of decrease in the Raman and fluorescence signals. Hence, we instead used the fluorescence signal, which is the featureless background, to extract the photo-bleaching rate. Control experiments showed that PMMA, graphene, and APTES gave no fluorescence signal (Figure 3, we also note that in these control samples, no Raman peaks of APTES and PMMA were observed due to the short integration time and the low Raman cross section), ensuring that the fluorescence decay is entirely due to the photo-bleaching of the dye. At the beginning of the experiment, the fluorescence intensity of FITC is lower in the presence of graphene than in the absence of graphene, which is consistent with graphene-induced fluorescence quenching. However, at the end of the experiment, the fluorescence intensity of FITC in the presence of graphene is higher than that in the absence of graphene, clearly demonstrating that graphene indeed slows down the photo-bleaching of the dye.

**Figure 4:**
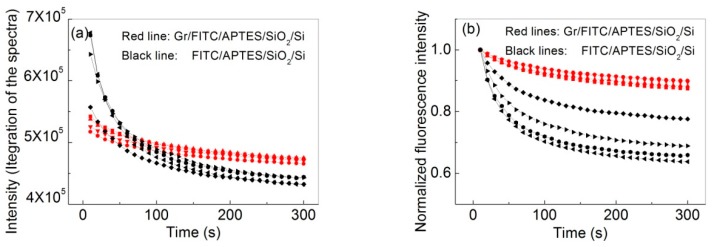
The photo-bleaching curve of FITC dye contacted with graphene (red) and without graphene (black). The intensity was calculated by integration from 583 to 2411 cm^−1^ (**a**) and normalized by the maximum value (**b**). Each curve represents a separate kinetic measurement on a different location of the same sample. Each spectrum was integrated for 5 s and measured every 10 s over a period of 5 min.

Figure 4 shows the typical kinetics of photo-bleaching of the FITC dye. Each curve represents a separate measurement on a different spot of the same sample. There is a ca. 20% variation in the initial fluorescence intensity, which we attribute to the random variation in the density of the adsorbed dye. To better visualize the data, the integrated fluorescence intensity is normalized by the maximum intensity after background correction (Figure 4b). The curves in Figure 4b are best fitted by a biexponential decay function (Equation (1)).

I (*t*) = α_1_*exp(−*k*_1_*t*) + α_2_*exp(−*k*_2_*t*) + I_0_ (1)

Here, the offset I_0_ is attributed to the dark current of the Raman CCD (charge-coupled device) camera, and α and *k* are the pre-exponential factors and photo-bleaching rate, respectively. The double exponential behavior suggests that two parallel pathways are contributing the photo-bleaching of the dye and/or the presence of two populations of dyes in different photochemical environments. The reason behind the double exponential behavior will be studied in a future work. 

The fast and slow components of the biexponential decay are 0.049 ± 0.009 s^−1^ and 0.010±0.002 s^−1^ in the absence of graphene and are 0.044 ± 0.007 s^−1^ and 0.0060 ± 0.0006 s^−1^ in the presence of graphene (Table 1). Thus, the presence of graphene reduced the bleaching rate for both kinetic populations. We also find that the presence of graphene also increases the ratio of the slow component in the overall decay. Specifically, the ratio α_2_/(α_1_ + α_2_) increased from ca. 0.4–0.5 in the absence of graphene to ca. 0.7 in the presence of graphene. At the same time, the rate constant for the slow decay decreased by 40% (from 0.01 s^−1^ to 0.006 s^−1^). Overall, the average photo-bleaching rate of dye in the presence of graphene is decreased by 46.3% (from 0.011 s^−1^ to 0.0063 s^−1^) compared to that in the absence of graphene. The average photo-bleaching rate $\bar{k}$ was calculated using the following equation [22].

(2) $$\bar{k}=\frac{\alpha_{1}/k_{1}+\alpha_{2}/k_{2}}{\alpha_{1}/k_{1}^{2}+\alpha_{2}/k_{2}^{2}}$$

### 3.3. Mechanism of Reduced Photo-Bleaching

**Figure 5:**
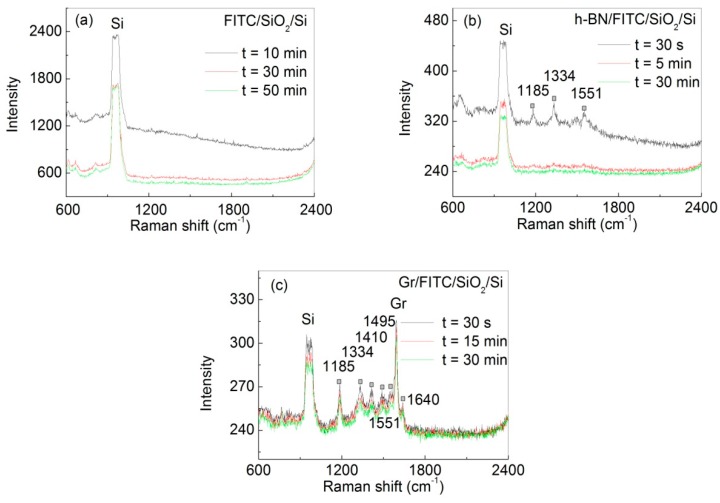
Raman spectra of FITC dye molecules in different structures: (**a**) FITC/SiO_2_/Si (Raman integration time was 10 min with 10 min delay between measurements); (**b**) h-BN/FITC/SiO_2_/Si (Raman integration time was 30 s with 30 s delay between measurements); and (**c**) Gr/ FITC/SiO_2_/Si (Raman integration time was 30 s with 30 s delay between measurements). Raman was measured at the same location in each sample. Peaks marked with “^■^” are from the FITC dye. Note that the SiO_2_ substrates used here were not modified with APTES, which results in lower surface coverage of FITC dye.

Previous studies in this area of research suggest that graphene protects the dye from photo-bleaching by isolating it from air [12]. To test this hypothesis, we compared the photo-stability of FITC dye in the presence and in absence of graphene or h-BN under continuous laser exposure. The Raman data in Figure 5 clearly shows that graphene prevents the dye from photo-bleaching. For FITC dye adsorbed on SiO_2_/Si without graphene (Figure 5a), strong fluorescence background is observed at the beginning of the experiment, which buried the Raman signals. The fluorescence signal completelyα pre-exponential factor, *k* photo-bleaching rate, and $\bar{k}$ average photo-bleaching rate disappeared after 30 min of continuous laser illumination. For FITC dye sandwiched between h-BN and SiO_2_/Si (Figure 5b), both fluorescence background and Raman signal are clearly observed at the beginning of the experiment. However, both the Raman peaks and the fluorescence disappeared 5 min later. Here, the observed Raman peaks are attributed to the h-BN-induced Raman enhancement [23]. In contrast, for FITC sandwiched between graphene and SiO_2_/Si (Figure 5c), the Raman peaks are clearly observed. The very different behavior between h-BN and graphene shows that merely having a gas barrier film (e.g., h-BN) is not sufficient to protect the dye from photo-bleaching [24]. It is also worth noting that in this experiment we did not modify the SiO_2_ substrate with APTES, which should reduce the surface coverage of FTIC dye compared to the experiment shown in Figure 2 and Figure 4. In this case, we observed lower FL background (e.g., compare Figure 5c with Figure 2D) and slower bleaching kinetics (e.g., compare Figure 5c with Figure 4b), which we attribute to a better physical contact between the dye and graphene at lower surface coverage.

The photo-bleaching of organic dye is commonly attributed to oxygen, which exists in a ground state triplet (^3^O_2_) and is an efficient quencher for a number of fluorescence dyes [25]. The dye molecules in the excited triplet state (T*) undergo permanent photochemical destruction during the photo-bleaching process. A photo-bleaching mechanism via the reaction between an excited triplet state (T*) dye molecule and an oxygen molecule (D-O reaction) has been reported [26]. Quenching of T* by ^3^O_2_ leads to the formation of semi-oxidized (X) radical form of the dye and HO_2_ or O^2−^. By adding a barrier layer between oxygen and dye, the D-O reaction will be effectively prevented, thus reducing the subsequent photo-bleaching and photodecomposition process. If D-O reaction is the predominant mechanism, similar observation will be found when the graphene was substituted by other oxygen diffusion barrier layers, such as h-BN. However, this mechanism is contradicted by the results from h-BN/FITC/SiO_2_/Si sample, as shown in Figure 5b. h-BN is known as an excellent oxygen barrier [24]. In our experiments, we found that the FITC dye decomposed faster in the presence of h-BN than in the presence of graphene, indicating the decrease of photo-bleaching rate of FITC dye by graphene is not mainly caused by the physical separation between the dye and oxygen. Instead, we suggest that the difference in the electronic properties of graphene and BN is playing a role. Graphene is a good electron conductor, in contrast to h-BN, which is an electronic insulator [9,17]. The anti-photo-bleaching of the dye molecule in the presence of graphene will be more effective in the presence of free conduction electron in graphene, as discussed below.

Quenching of fluorescence of dye could occur through either charge transfer or energy transfer and, in the latter case, two pathways are known: the Förster resonance energy transfer and the surface energy transfer [22,27]. The Förster resonance energy transfer is through the interaction of dipole-dipole while the surface energy transfer is through the interaction of dipole and surface-free electron in metallic materials. Both charge transfer and energy transfer can result in fluorescence quenching of the dye by graphene, which is observed in our work and consistent with previous reports [22,27]. While both mechanisms will result in a reduced fluorescence life time, the two pathways show different distance dependence and the charge transfer will also result in a transient change in the charge state of the dye. Unfortunately, our current data cannot distinguish the two quenching mechanisms and further studies using time-resolved methods may be needed to clarify the detailed mechanism.

It is worth comparing our result with that of Zhao et al., who reported that the photochemical stability of dyes increases when they are adsorbed underneath graphene compared to those adsorbed above grapheme [12]. In their work, it was proposed that graphene acts as a barrier for oxygen to enhance the photochemical stability of the dyes. A major difference between the work of Zhao et al. and the current study is the substrate: Zhao et al. used Ag while we used SiO_2_/Si. It is likely that the dyes already experience significant quenching by the Ag substrate, and therefore the photochemical stability can no longer be enhanced further by the quenching provided by graphene. In this case the effect of barrier film becomes important. In contrast, the dyes in our experiments rely on graphene to reduce their lifetime at the excited state. In this case, the difference between graphene and h-BN becomes significant. Although both are good gas barriers, the unquenched dyes may still be oxidized by the oxygen or other impurities trapped between graphene or h-BN and the SiO_2_. In this case, the better quenching efficiency of graphene, as can be seen by comparing the fluorescence background of the dye in the presence and absence of graphene (Figure 5b,c), plays a major role in determining the photochemical stability of the dye. Thus, our result complements and extends that reported in Zhao et al., highlighting the importance of both graphene and the supporting substrate in understanding the overall photochemical stability of organic dyes. 

Finally, we comment on the limitations of our study. We chose to use FITC in our kinetic study because this dye can be covalently attached to the SiO_2_ surface via APTES, ensuring that no more than a monolayer amount of dye is present.For other dyes, we observed inconsistent fluorescence quenching behavior (Figure 1), which we attribute to multilayer adsorption of dyes.Therefore, our conclusion is specific to FITC. Work is in progress to study the photobleaching behavior of a broader range of organic dyes in the presence of graphene.

## 4. Conclusions

In summary, we have showed that CVD-grown graphene can be used as a substrate to enhance the Raman signal of a wide range of fluorescence dyes. By comparing graphene with h-BN, we showed thatalthough both are good barrier films for oxygen, graphene is much more effective in improving the photostability of FITC. Therefore, the energy/charge transfer plays an important role in improving the photochemical stability of the FITC dye sandwiched between SiO_2_ and graphene.

## Figures and Tables

**Table 1:** The photo-bleaching rate and pre-exponential factor of the biexponential decay obtained by fitting the photo-bleaching curve of FITC dye in the absence (black) and presence of graphene (red) in Figure 4. Note that experiment #4 of Gr/FITC/APTES/SiO_2_/Si sample showed extremely slow photo-bleaching kinetics (slower than in the absence of graphene) and was not included in our analysis. It is possible that this data was taken from an area with aggregation of dyes or overfoldedgraphene.

Sample Structure		*α*_1_	*k*_1_ (s^−1^)	*α*_2_	*k*_2_ (s^−1^)
FITC/APTES/SiO_2_/Si	test area 1	0.60	0.037	0.70	0.0078
	test area 2	0.90	0.053	0.54	0.0097
	test area 3	0.71	0.048	0.66	0.0088
	test area 4	0.81	0.059	0.64	0.0119
	average	0.8 ± 0.1	0.049 ± 0.009	0.64 ± 0.07	0.010 ± 0.002
	$\bar{k}$(s^−1^)	0.011 ± 0.002			
Gr/FITC/APTES/SiO_2_/Si	test area 1	0.39	0.037	0.90	0.0064
	test area 2	0.30	0.045	1.0	0.0063
	test area 3	0.43	0.050	0.99	0.0054
	test area 4 (discarded)	0.6	0.022	1.1	0.0023
	average	0.37 ± 0.07	0.044 ± 0.007	0.96 ± 0.05	0.0060 ± 0.0006
	$\bar{k}$(s^−1^)	0.0063 ± 0.0006			

## Supplementary Materials

The following are available online at http://www.mdpi.com/2079-4991/7/10/337/s1.
