# Supplementary material for: Raman Enhancement and Photo-Bleaching of Organic Dyes in the Presence of Chemical Vapor Deposition-Grown Graphene
Source: Nanomaterials (Basel). 2017 Oct 19;7(10):337. doi: 10.3390/nano7100337 (PMC5666502; doi:10.3390/nano7100337)
Supplement: Supplementary file 1 [file nanomaterials-07-00337-s001.pdf]

Supporting Information

Raman Enhancement and Photo-Bleaching of Organic Dyes in the Presence of Chemical Vapor Deposition-Grown Graphene

Jiaxin Weng, Shichao Zhao [1,2,*], Zhiting Li [2], Karen B. Ricardo [2], Feng Zhou [2], Hyojeong Kim [2] and Haitao Liu [2,*]

[1] College of Materials & Environmental Engineering, Hangzhou Dianzi University, Hangzhou 310018, China
[2] Department of Chemistry, University of Pittsburgh, Pittsburgh, Pennsylvania, PA 15260, USA
* Correspondence: zhaoshichao@hdu.edu.cn (S.Z.); hliu@pitt.edu (H.L.)

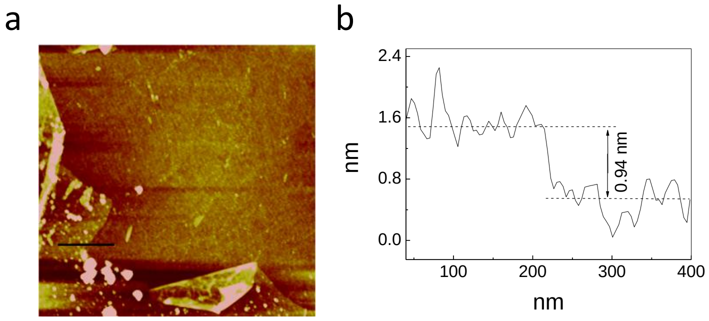

Figure S1. (**a**) Topographic AFM and (**b**) cross section of a graphene piece on a Gr/FITC/APTES/SiO_2/Si sample. The sample was washed in acetone for 12 h. The height of the single layer graphene was higher than the expected 0.34 nm, which is well documented in the literature [1].

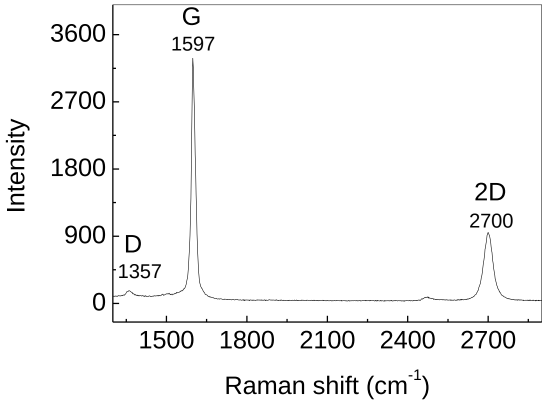

Figure S2. Raman spectrum of a single layer graphene transferred on the SiO_2/Si substrate.

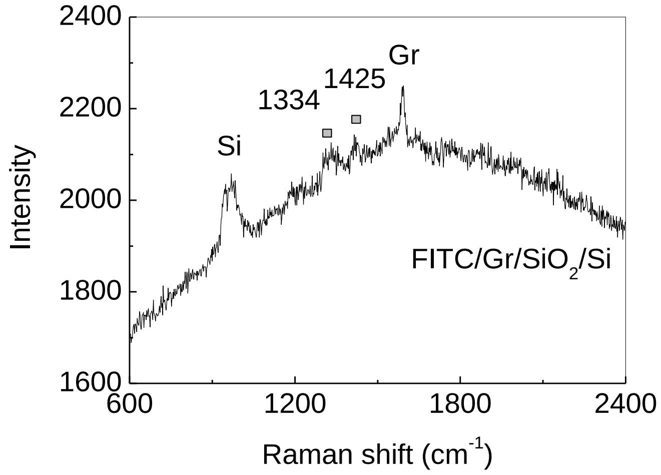

Figure S3.Raman-FL spectra of FITC adsorbed on the surface of grapheme (FITC/ Gr/SiO_2/Si).

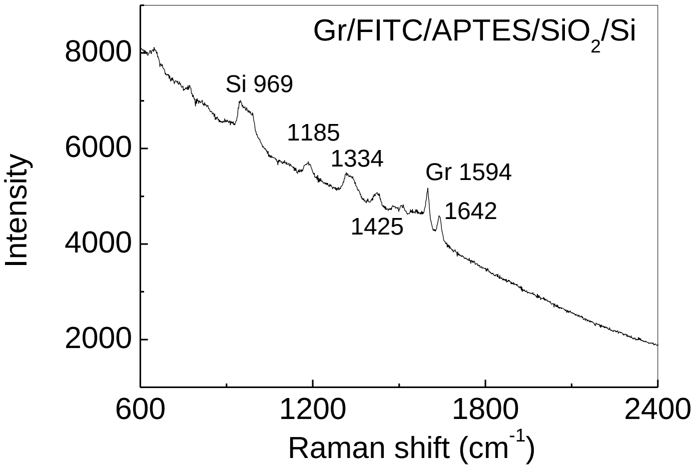

Figure S4. Raman-fluorescence spectra of FITC in the presence of graphene (Gr/FITC/APTES/SiO_2/Si). The sample was the same one in Figure 2b. The spectrum was integrated for 10 min.

Reference

1. Zhao, S.C.; Surwade, S.P.; Li, Z.T.; Liu, H.T. Photochemical oxidation of CVD-grown single layer graphene. *Nanotechnology***2012**, *23*, 355703.
